# An Effective Algorithm to Find a Cost Minimizing Gateway Deployment for Node-Replaceable Wireless Sensor Networks

**DOI:** 10.3390/s21051732

**Published:** 2021-03-03

**Authors:** Sun-Ho Choi, Yoonkyung Jang, Hyowon Seo, Bum Il Hong, Intae Ryoo

**Affiliations:** 1 Department of Applied Mathematics and the Institute of Natural Sciences, Kyung Hee University, Yongin 17104, Korea; sunhochoi@khu.ac.kr (S.-H.C.); hyowseo@khu.ac.kr (H.S.); bihong@khu.ac.kr (B.I.H.); 2 Department of Computer Science and Engineering, Kyung Hee University, Yongin 17104, Korea

**Keywords:** wireless sensor networks (WSNs), internet of things (IoT), optimal gateway deployment, divide and conquer

## Abstract

In this paper, we present an efficient way to find a gateway deployment for a given sensor network topology. We assume that the expired sensors and gateways can be replaced and the locations of the gateways are chosen among the given sensor nodes. The objective is to find a gateway deployment that minimizes the cost per unit time, which consists of the maintenance and installation costs. The proposed algorithm creates a cost reference and uses it to find the optimal deployment via a divide and conquer algorithm. Comparing all cases is the most reliable way to find the optimal gateway deployment, but this is practically impossible to calculate, since its computation time increases exponentially as the number of nodes increases. The method we propose increases linearly, and so is suitable for large scale networks. Additionally, compared to stochastic algorithms such as the genetic algorithm, this methodology has advantages in computational speed and accuracy for a large number of nodes. We also verify our methodology through several numerical experiments.

## 1. Introduction

Wireless sensor networks (WSNs) consist of sensor nodes and sinks. The sensor nodes detect physical phenomena and transmit all collected data to a gateway called a sink in a multi-hop manner according to a designated routing protocol [1]. These WSNs are used in many areas. For example, collecting and transmitting data by applying internet of things (IoT) technology makes it easier to manage the power transmission system located in a dangerous terrain [2]. In addition, sensor networks are applied in various areas, including human health monitoring [3], home IoT [4], smart factories [5], environmental and weather information measurement [6], stability measurement of buildings [7,8], and control of various smart devices [9].

The sensor-based IoT is regarded as one of the key technologies of the Fourth Industrial Revolution. Since the fields that utilize sensor-based IoT are expected to increase further [10] and the network size also increases [11], the need for optimizing gateway development emerges [12]. Effective deployment of gateways is also important for the stability of the entire sensor network [13]. If the gateways are distributed too sparsely, individual sensors may not be able to transmit data to the gateway after sensing it, which leads to propagation latency or data loss. On the other hand, if the gateways are dense, then the lifespan of the network is reduced.

This study deals with an efficient way to find a gateway deployment to minimize the total cost of a system with a large number of nodes. For a given sensor node topology, we can choose the gateway locations among the sensor nodes. In general, gateways are more powerful than sensor nodes, while installation and maintenance costs are more expensive than sensor nodes [14]. Therefore, determining the number and location of gateways greatly affects the overall cost and performance of the network. Much literature on optimal gateway deployment attempts to find the longest lifetime deployment for a determined number of sensors and gateways [15]. However, due to recent advances in various delivery technologies and the miniaturization of sensors, it is easy to replace expired sensors with new ones. This process can be expressed as a function of cost. Therefore, to minimize the cost of the entire network, it is reasonable to consider the cost per unit time rather than maximizing the lifetime of the network with non-replaceable nodes. Additionally, we consider the gateway as a terminal to simplify our problem, and the communication cost between terminal points will not be considered. See [14] for related issues.

In this graph model, the simplest way to find the optimal gateway deployment is by searching all possible locations of the gateways and comparing their costs of all cases under the connectivity constraints. From this, we can obtain the optimal gateway placement for a given topology. However, this method takes a lot of computation time. For example, if we use a personal computer to compare the costs of all possible cases for 30 sensor nodes, it would take about 3663 days, or about 10 years. Therefore, it is practically impossible to enumerate all possible cases on a large number of nodes.

We propose a simple iterative algorithm to find a sufficiently efficient gateway deployment in a short time. The main idea is to consider subgraphs and cost references. In the optimization point of view, by considering cost reference for the subgraphs, we can get the optimal cost. Therefore, it is easily applicable to the industry. The entire network is processed in four steps. We find the optimal location of the gateway by dividing the network based on the communication distance in the first step and the cost in the second step. In the third step, we use a cost reference and some kind of puzzle-solving method to find an effective deployment. After the third step, if there is a sub-network in which one gateway takes charge of sensor nodes higher than a certain level, then additional gateways are placed in the sub-network.

We note that stochastic methodologies have been widely used in recent years. However, they have a large fluctuation in the speed and accuracy of calculation depending on the network topology, and their computation time is proportional to the square of the number of nodes and the number of generations. Considering various realistic constraints also increases the amount of computation cost. In addition, there is a problem of finding an appropriate random parameter for each topology, which may make it difficult to apply these kinds of methodologies to practical problems.

However, the accuracy and calculation speed of our methodology are relatively uniform regardless of the shape of the topology and the amount of computation is proportional to the number of nodes. Therefore, our methodology is suitable to be applied to a network with a large number of nodes. To verify this, we provide comparisons between the genetic algorithm and our methodology. Moreover, in our algorithm, we compute the cost reference only once. We can then use it repeatedly for different topologies to reduce the computation time.

The rest of this paper is organized as follows: In Section 2, we provide a brief review of the research related to gateway deployment. In Section 3, we define a cost function and generate a cost reference. The proposed method for gateway deployment is described in Section 4. In Section 5, we compare the suggested method in this paper and existing methods through simulations. Finally, in Section 6, we complete the paper with a discussion and conclusions.

## 2. Related Works

As the field of application of WSNs is diverse, WSNs of various structures are utilized. For this reason, various gateway deployment methods have been proposed. They are broadly divided into evolutionary and deterministic methods. Here, we review genetic algorithms and recursive algorithms for the evolutionary methodology and degree-based algorithms and linear programming for the deterministic methodology.

### 2.1. Evolutionary Algorithms

The genetic algorithm is one of the most frequently used methods for optimization problems. For example, the genetic algorithm-based self organizing network clustering (GASONeC) method is used for selecting optimal cluster headers among sensor nodes. In this selection process, GASONeC considers the number of sensor nodes and the distances between the selected nodes and the remaining sensor nodes as fitness function variables [15]. It also can be applied to gateway deployment problems assuming that the energy of the selected node is infinite. Its methodology is simple so that it can be easily applied to a variety of problems. However, for each calculation, this method may not get the same result, since it uses a random probability to select the location of the gateways. Moreover, as the number of sensor nodes increases, the complexity is increased significantly.

The genetic algorithm for hop count optimization (GAHO) also uses the genetic algorithm to minimize data latency which causes data transmission delay and increases the possibility of packet loss [16]. The GAHO divides the entire network into clusters, and deploys a gateway for each cluster. The genetic algorithm is applied to divide the entire network into clusters. After calculating the central location of each cluster, it is used as the temporary location of the gateway. The area around the temporary location is divided into several grid areas. The gateway is placed in the grid cell that has the maximum number of sensors. In [16], the genetic algorithm is not used to find the location of the gateway, but only to form clusters. Here, one gateway must be unconditionally arranged for each cluster. Therefore, when the cluster configuration is not optimal, it is difficult to optimize the gateway arrangement.

In selective gateway and reinforcement learning-based routing (SGRL) method [17], a gateway deployment is proposed to increase the network stability between the gateways by avoiding route flapping. Moreover, taking into account the loss ratio, interference ratio and load at the gateways, this methodology selects the gateway deployment by using a reinforcement learning-based algorithm.

In [18], the authors consider the minimum set covering the problem and propose a recursive algorithm. This method is the most similar to our algorithm. Each sensor has its own transmission range because of its lifetime. The algorithm contains three main parts: decomposition, multiple search, and compression. In the decomposition step, it finds the covering that covers all sensors and finds duplicated sets that cover the same sensor in the multiple search step. Finally, the over installed gateway is eliminated in the compression step. This method repeats the process until the algorithm finds the minimal coverings.

### 2.2. Deterministic Algorithms

The gateway deployment problems can be formulated as a maximization problem for the objective function with constraints. Linear programming (LP) or integer linear programming (ILP) are used to find the optimal locations for gateways.

In the heuristic internet gateway (H-IGW) placement method [19], deploying gateways is treated as a linear program issue and two algorithms are proposed for cost-effective gateway deployment. Degree-based greedy dominating tree set partitioning (GDTSP) sets the node with the highest connection degree as a gateway, and weight-based GDTSP sets the node with the most paths from all other nodes as a gateway. The two algorithms complement a large amount of computation for deploying gateways to large-scale networks using linear formulations. The H-IGW placement method assumes that there is no power constraint. For a given natural number R ∈ N, this method divides the entire graph into subgraphs with an R-hop diameter, and places the gateway for each subgraph by selecting degree-based or weighted-based GDTSP.

The ILP model is used in [20] to find the optimal gateway placement when the number of gateways is fixed. In [14], the objective function is set up to minimize installation cost and the number of overload critical nodes with coverage flow and balance constraints. The authors used an ILP model and they also proposed a heuristic algorithm to tackle large scale networks. See also [21] and its references for numerous literatures dealing with ILP or LP problem.

The multiple-gateway deployment (MGD) method deploys gateways where the average distance between the nodes and the gateways is minimized [22]. The distance is calculated based on the hop count and the expected number of transmissions. In the MGD method, the target network is regarded as a connected graph G(V,E) and is divided into k groups that have the same number of vertices. Then this method formulates k connected trees from k groups and selects a gateway for each tree to minimize the sum of the distance between the gateway and the rest nodes on each tree.

The zero degree (ZD) algorithm method aims to increase efficiency by minimizing the number of gateways to reduce the total number of clusters and the number of hops between the gateway and the other nodes [23]. A gateway is selected by the ZD-S algorithm or the ZD -L algorithm. The ZD-S algorithm selects the node with the fewest general nodes that can be reached with one hop as a gateway, and the gateways are placed close to each other. The ZD-L algorithm selects the node with the highest connectivity with the neighboring nodes as a gateway, and the gateways will be located far from each other.

These gateway deployment methods take a lot of time to get a solution. Evolutionary algorithms spend a little time relatively, but because they are based on probability, the solution obtained is often not optimal. Also, the solution is unreliable because the result is different each time the algorithm is applied to the same network topology. The method proposed in this paper shows near-optimal results while consuming a short time by using divide-and-conquer algorithm. The results are reliable because they are always the same every time when this method is applied for the same network topology.

## 3. Network Model and Cost Function

In this paper, we find the most cost-effective gateway deployment for a given network topology. The two dimensional and single-tier multi-hop network is considered. We assume that for the fixed flat architecture network, the collected data in some sensor node are relayed to a gateway as the terminal base station and there is no communication between gateways. In addition, there are many factors regarding cost and efficiency. The other settings are described as follows:

(1) Sensor nodes and gateways are replaced when their lifetimes are over. Thus, repetitive reinstallation costs occur.
(2) The maintenance cost depends only on the number of hops between nodes and gateways.
(3) For a given sensor node network, we choose the gateway locations among the sensor. nodes.
(4) Efficiency is sufficient if the information from each node can be transmitted to at least one gateway.
(5) All sensors produce and transmit the same amount of information with the same frequency, and consume the same amount of energy each time they transmit information.
(6) The depreciation costs are incurred in proportion to the amount of information transmitted. Then the energy consumption and depreciation cost of each node and gateway depend on the number of hops between the nodes and gateways.

Next, we need to define the cost function. The cost function reflects the energy consumed and the lifespans of the sensor nodes and gateways. For a given set of sensor nodes $V_{s}=\left\{x_{1},\;\ldots ,\;x_{n}\right\}$ and gateway nodes $\;V_{g}=\left\{y_{1},\;\ldots ,\;y_{m}\right\}$, the entire network is represented by a graph $G\left(V,E\right)$ consisting of the set of edges E connecting each node and the set of vertices $V=V_{s}\cup V_{g}=\left\{x_{1},\ldots ,x_{n},y_{1},\ldots ,y_{m}\right\}$. For each sensor node $x_{i}$, only one gateway for receiving information will be assigned We denote the assigned gateway of $x_{i}$ by $f(x_{i})$. Thus, f is a function from $V_{s}$ to $V_{g}$. In the graph $G\left(V,E\right)$, if the minimum number of hops connecting two nodes v_i_ and v_j_ is expressed as H[v_i_,v_j_], the sum of energy consumption and depreciation cost of all sensor nodes is given by:

(1) $${\mathrm{Cos}t}_{s}=\sum_{i=1}^{n}c_{\mathrm{se}}\left(H\left[x_{i},f\left(x_{i}\right)\right]\right).$$

where $c_{se}$ is a function that expresses the sum of the average energy consumption cost of the sensor node for each hop per unit time, and the average depreciation cost of one sensor node over its lifespan. Here, $c_{se}$ contains the installation cost. In a similar way, the set of sensor nodes connected to gateway *y_i_* is simply expressed by $f^{-1}\left(y_{i}\right)$. Therefore, for number of gateways m∈ℕ, the sum of energy consumption and depreciation costs of all gateways is given by:

(2) $${\mathrm{Cos}t}_{g}=m\;c_{gi}+\sum_{j=1}^{m}\sum_{x\in f^{-1}\left(y_{j}\right)}c_{ge}\left(H\left[y_{j},x\right]\right),$$

where $c_{ge}$ is a function that express the sum of the average energy consumption cost for each hop count of the gateway per unit time and the average depreciation cost of one gateway over its lifespan, and $c_{gi}$ is the installation cost of one gateway. In the cost function, to apply this method to real situation, the parameters $c_{se}$, $c_{ge}$, and $c_{gi}$ are calculated by a statistical method. For example, the estimated values for $c_{gi}$ is obtained by dividing the installation cost by the average usage time.

The entire topology can be divided into several subgraphs centered on each gateway. The total cost for the entire topology is the same as the sum of the subgroup costs, since only one gateway will be assigned for each sensor node. Thus, if our partitioning is proper and we find a deployment for the minimum cost in each subgraph, then we can obtain the total minimum cost for the entire topology. This method has the advantage of shortening the computation time. Finding the optimal placement for the whole network takes a lot of time, but repeatedly finding the optimal placement for the subgraphs does not take much time. However, if it is not properly divided into subgroups, there may be a lot of differences from the ideal optimal cost. Therefore, we need to divide the groups appropriately to obtain a deployment close to the ideal optimal cost in a short time.

**Figure 1:**
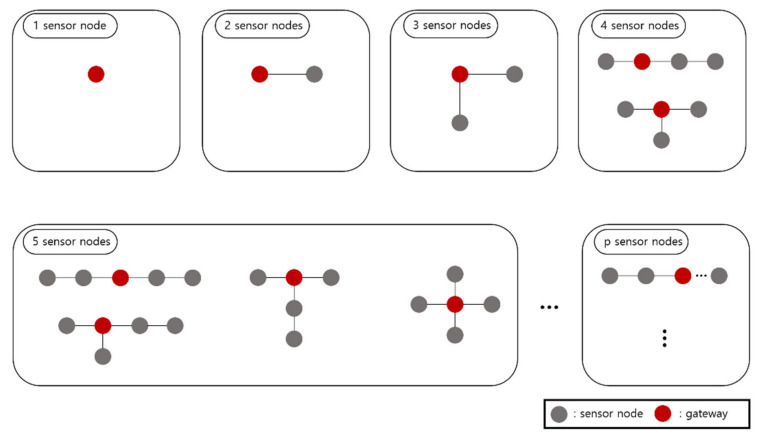
Example of the cost reference.

We next create a cost reference using the Equations (1) and (2). The cost reference is information for all topology cases that can be generated for each number of sensor nodes and the location of the gateway that minimizes the cost in a given topology. We let p be the gateway capacity, i.e., the maximum number of sensor nodes that can be connected to one gateway is p. Then, the cost reference has information from 1 to p sensor nodes. Figure 1 is an example of the cost reference. The gray circles are sensor nodes and red circles are gateway locations that minimize cost.

In Figure 1, in the case of one sensor node, it is only the case that the corresponding sensor node is set as a gateway. In the case of two sensor nodes, there is only one topology, and there is no difference in cost when either of them is designated as a gateway. In the case of three sensor nodes, the topology is one, and it is optimal that the sensor node at the center is designated as a gateway. In the case of 4 sensor nodes, there are two topology cases, and the ideal location of the gateway for each topology is marked with red in Figure 1. In the case of 5 sensor nodes, the number of topology cases is 4, and the optimal gateway designation position for each topology is also marked with a red gateway. As above, all topology cases from 1 to p sensor nodes and the optimal gateway location for each case can be found and stored in the cost reference.

## 4. Divide and Conquer Algorithm with a Reference Deployment Set

The most obvious way to find the optimal gateway deployment is to compare the total costs of all possible cases. The number of gateways that can be installed here ranges from one to the total number of sensor nodes. This method takes into account all possible gateway deployments, so the optimal result must be obtained. However, as the number of sensor nodes increases, the computational amount increases exponentially. Therefore, when the number of nodes is rather large, it is almost impossible to obtain the optimal gateway deployment by this method. In this section, we propose an efficient method to obtain the optimal gateway deployment in a short time for use in real industries. The proposed method is a kind of ‘divide and conquer’ algorithm.

Our method is divided into four steps. In the first and second steps, the sensor nodes are classified based on the communication distance and the cost function. In the third step, a gateway deployment is found by dividing the sensor nodes using the defined cost function. In the fourth step, if one gateway is connected to more sensor nodes than a given standard, a gateway is additionally arranged in the corresponding zone. We note that although this method does not achieve the smallest cost deployment in all cases, it is possible to obtain a sufficiently efficient gateway deployment in a short time. The GASONeC, GAHO, SGRL, H-IGW placement, MGD, and ZD algorithms, which have been studied for gateway deployment, also do not lead to an ideal optimal result, but they allow sufficiently effective placements in a relatively short time.

### 4.1. (Step 1) Node Classification Based on the Communication Distance

In this step, the entire network is divided based on the communication distance. Since data are transmitted in a multi-hop manner, we must install a gateway for each isolated node individually. Therefore, these kinds of nodes should be considered prior to gateway deployment.

**Figure 2:**
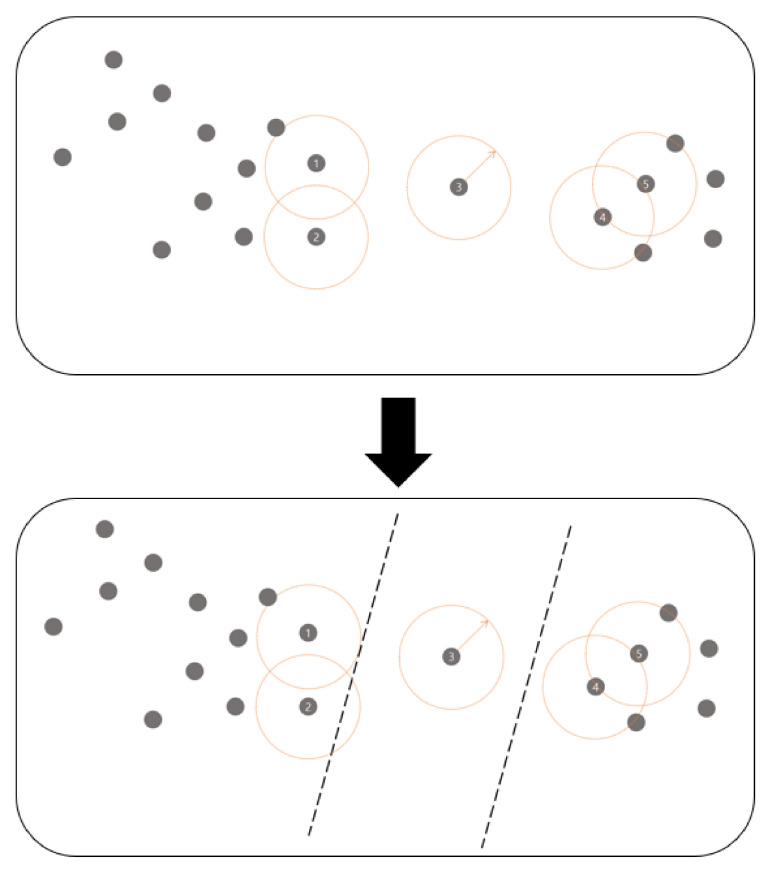
Division based on the communication distance in Step 1.

Figure 2 shows the methodology before and after going through the first step. The gray points represent the sensor nodes. In Figure 2, the orange dotted circles indicate the communication range of the corresponding sensor nodes. Sensors 1 and 2 cannot communicate with sensor 3. Therefore, sensor 3 must be set as a gateway for the connection constraint. With this motivation, in the first step, the network is divided into several subgraphs so that nodes within a communication range are defined as a group. For example, in Figure 2, it is divided into three groups: a group containing sensors 1 and 2, a group containing sensor 3, and a group containing sensors 4 and 5.

### 4.2. (Step 2) Node Classification Based on the Cost Function

In this step, the network is divided once more based on the cost function in the subgroup classified in the previous step. Here, the cost of each topology is calculated as the sum of Equations (1) and (2) defined in Section 3. From a given group obtained in Step 1, we generate an undirected graph looking for a combination of sensor nodes and gateways that make up the topology and minimizes its cost.

Let p be the maximum number of sensor nodes that can be connected to one gateway and q be the maximum number of gateways. Then we generate all sequences of numbers such that each number is less than p and the sum of all numbers in each sequence is the total number of the subgraph. Next, we remove the sequences that are longer than q. The numbers in the sequence will be one of the number of nodes in a subgroup. Then we can calculate the minimum cost for each sequence by referring to the cost reference obtained in the previous section.

**Figure 3:**
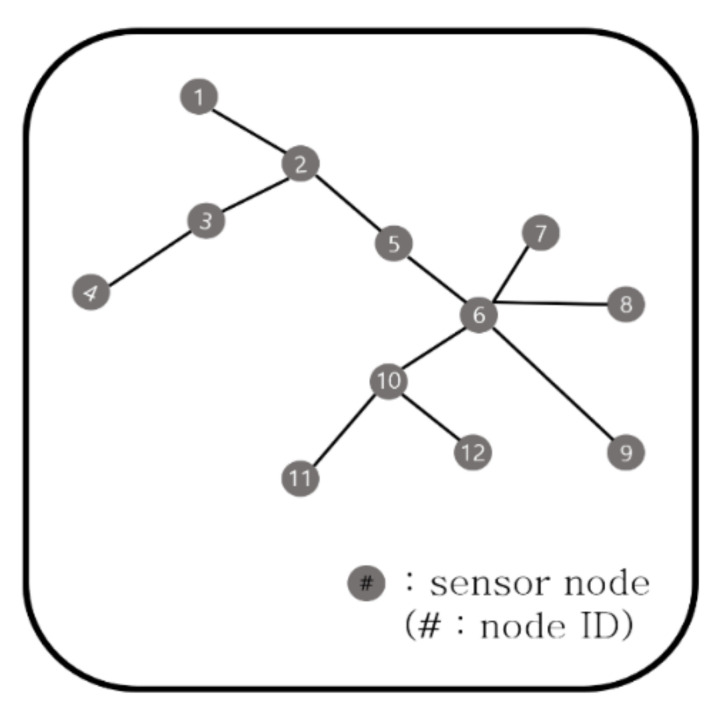
Generated tree from the subgraph after Step 1.

For example, Figure 3 shows the topology of the leftmost group after the first step in Figure 2. It consists of a total of 12 sensor nodes. When the maximum sensor node of each combination is *p* = 9 and the maximum number of gateway arrangements is q = 5, there are several cases in which 12 sensor nodes are divided. The smallest unit can be a combination of 12 groups with one sensor node, such as:

(1,1,1,1,1,1,1,1,1,1,1,1).

Next, it can be divided into combinations of two groups having one or two sensor nodes such as:

(2,1,1,1,1,1,1,1,1,1,1), (2,2,1,1,1,1,1,1,1,1), (2,2,2,1,1,1,1,1,1),

(2,2,2,2,1,1,1,1), (2,2,2,2,2,1,1), (2,2,2,2,2,2).

Combinations of one, two, and three sensor nodes are as follows:

(3,2,1,1,1,1,1,1,1), (3,3,1,1,1,1,1,1), (3,3,2,1,1,1,1),

(3,3,2,2,1,1), (3,3,2,2,2), (3,3,3,1,1,1), (3,3,3,2,1).

Similar to the above cases, we can obtain all possible combinations. Since the maximum number of gateways is 5, we remove the sequences that are longer than 5. For example:

(1,1,1,1,1,1,1,1,1,1,1,1), (2,1,1,1,1,1,1,1,1,1,1), (2,2,1,1,1,1,1,1,1,1), (2,2,2,1,1,1,1,1,1),

(2,2,2,2,1,1,1,1), (2,2,2,2,2,1,1), (2,2,2,2,2,2), (3,2,1,1,1,1,1,1,1), (3,3,1,1,1,1,1,1),

(3,3,2,1,1,1,1), (3,3,2,2,1,1), (3,3,2,2,2), (3,3,3,1,1,1), …

Then we calculate the lowest cost of each sequence and assign it to that sequence.

### 4.3. (Step 3) Topology Reconstruction

In this step, we use the combinations obtained in the previous step as puzzle pieces. Then we try to reconstruct the network graph by assembling the combinations. We fill the topology with the puzzle pieces in order beginning with the smallest cost. We have to check whether the subgraph can be reconstructed with the previous combinations or not, because the smallest cost case may not be constructed in the subgraph. Additionally, if an isolated sensor node occurs during reconstruction, the combination is corrected and applied again. Therefore, when an isolated sensor node occurs, the sensor node is necessarily included and divided accordingly.

**Figure 4:**
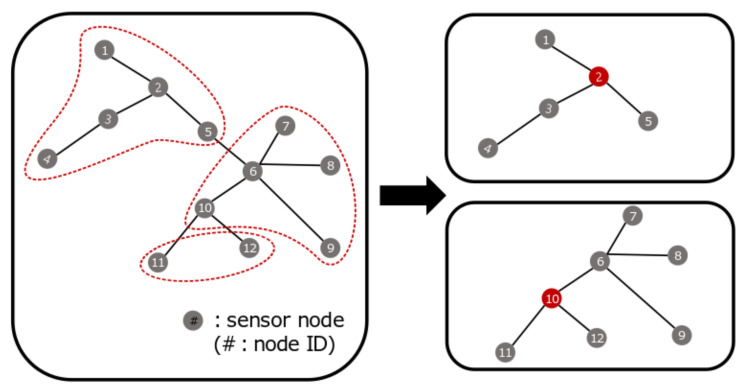
Division based on the cost function and optimal location of a gateway.

For example, Figure 4 begins by substituting the combination of (5,5,2), which is the least cost case in Figure 3, and finally results in reconfigurable (5,7). First, we start with sensor 1 and make it into one subgroup up to 5, and then we designate sensor 2 as a gateway by the cost reference. The cost is also calculated according to the cost reference. Thereafter, starting from sensor 7, the group is composed of 5 sensor nodes. If sensors 6, 7, 8, 9, and 10 are included in order, 11 and 12 are isolated. See the left figure in Figure 4. The red dotted line represents the groupings with the lowest cost. In this case, a total of seven sensor nodes are grouped, including sensor nodes that can be isolated. According to the cost reference, designating sensor 10 as a gateway is the minimum cost case.

### 4.4. (Step 4) Additional Arrangement

In this step (Algorithm 1), if one gateway is responsible for more than a certain number of sensor nodes in the previous result, the gateway is additionally placed in the corresponding zone. Here, the standard can be freely set by the administrator. When applying an algorithm to avoid isolated nodes in the previous step, too many nodes may be connected to one gateway. The reason why gateways are additionally deployed in this step is to compensate for this phenomenon.
**Algorithm 1.** Additional Arrangement1: Generate a cost reference ‘*C =* {$c_{1}$, $c_{2}$, ... , $c_{m}$}’ 2: ∀*^c^_i_* ∈ C, calculating the cost by sum of equation (a) and equation (b) defined in Section 3. 3: ∀*^c^_i_* ∈ C, save optimal position of gateway 4: the entire network is divided into ‘n’ subgraphs S = {$s_{1}$, ..., $s_{n}$} based on the communication distance 5: for i = 1, 2, …, n{ 6:    for j = 1, 2, …, q{ 7:    while(find minimum sum of equation (a) and equation (b)) { 8:     divide $s_{i}$ into subgraphs $g_{a}$, 1 ≤ a ≤ q with |$g_{a}$| ≤ p. 9:    } 10:  } 11: } 12: if (there is isolated sensor node){ 13:    include that sensor node to $g_{a}$ 14: }m: the number of cost reference p: capacity of gateway q: the maximum number of gateway arrangements

## 5. Numerical Simulations

In this section, we will numerically determine the optimal gateway deployment using the described method in Section 3 and Section 4. By comparing all possible cases, the optimal gateway deployment for a given topology can be obtained. We first compare the times for the method presented in this paper and the method considering all cases. The time refers to the time spent to obtain a solution for deploying gateways by applying the methodology. Next, we present the numerical results to compare the genetic algorithm and our algorithm. As we pointed out in the previous section, the proposed algorithm may not give the optimal gateway placement but provides a result sufficiently close to the optimal. Therefore, to check the validity for the total cost, we compare the proposed method with the method checking all cases that spends much time to obtain the result but gives the exact optimal placement for the gateway. By comparing the results obtained from the proposed method and checking all cases, we can check that how close the proposed method to optimal placement. A genetic algorithm is a comprehensive algorithm for graphical models. Therefore, we compare the proposed method with the genetic algorithm in terms of cost and computation time.

### 5.1. Comparison with the Method to Check All Cases

For simulation, we use Matlab and set the cost functions as follows:

(3) $$c_{\mathrm{se}}\left(x\right)=3+\sqrt{x},{\;c}_{\mathrm{gi}}=10,{\;c}_{\mathrm{ge}}=\sqrt{x},\;p=9,\;q=5.$$

In our methodology, when one gateway is responsible for more than half of the entire topology in the previous result, the gateway will be additionally placed in the corresponding zone. The topology is randomly generated by increasing the number of sensor nodes from 11 to 18 by one. Table 1 and Table 2 show the averages of the time taken to find the gateway deployments for five topologies per number of sensor nodes. Table 1 is the result of finding the optimal gateway layout among all cases, and Table 2 is the result of applying the method proposed in this study to find the optimal gateway layout.

**Figure 5:**
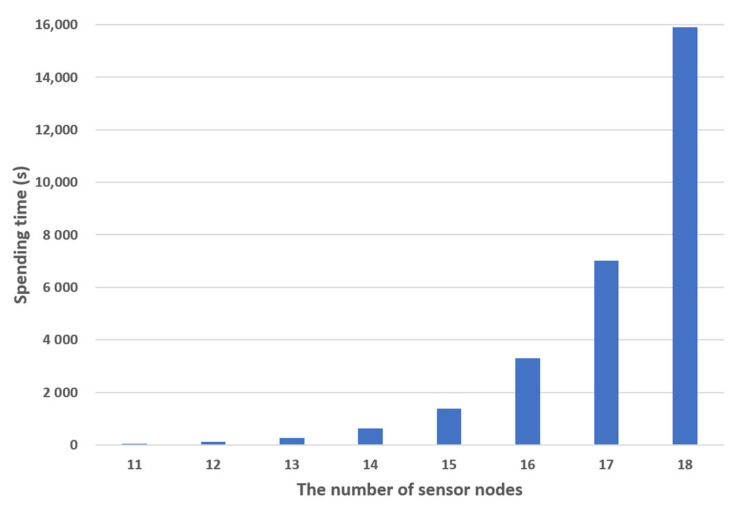
Average spending time to check all cases.

In the case of Table 1, whenever a sensor node is added, it takes about 2.28 times more time to find the gateway deployment. Figure 5 shows the average spending times for the method to check all cases. These results show that the spending time increases exponentially as the total number of nodes increases. When calculating for 21 sensor nodes, it takes about 2.2 days. This is expected to take about 10 years when targeting 30 sensor nodes. Therefore, it is impossible to obtain a gateway arrangement by analyzing virtually all cases.

**Figure 6:**
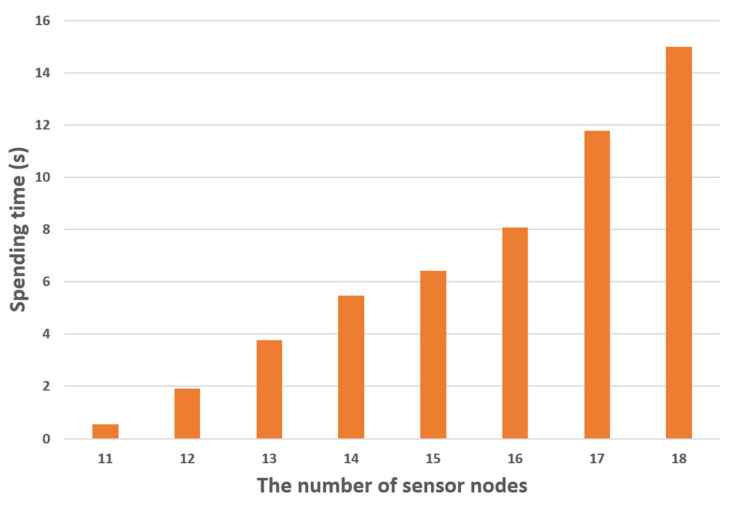
Average time spent by the proposed method.

In the case of Table 2, as the number of sensor nodes increases, the time required increases by about 1.95 s. Figure 6 shows the average spending time for the proposed method. This result shows that the spending time increases linearly as the total number of nodes increases. Even when targeting 30 sensor nodes, it takes about 24 s.

**Figure 7:**
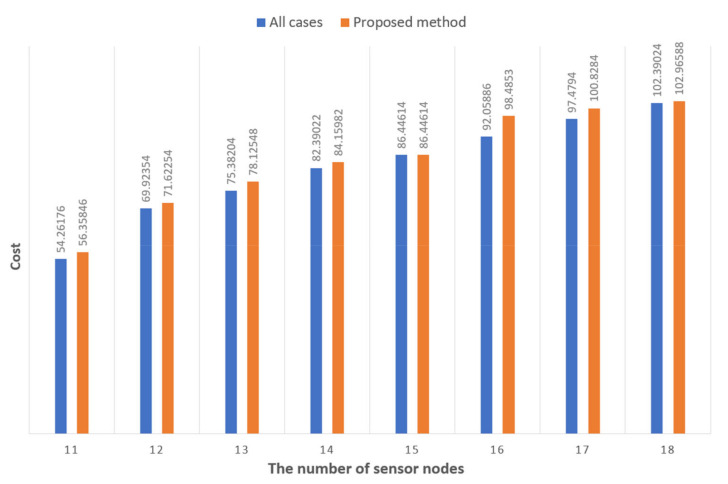
Comparison of the costs in two methods.

Figure 7 compares the costs in the method to check all cases and the method proposed. In Figure 7, it can be seen that there is little difference between the two methods in terms of the cost. In addition, when considering the optimal gateway placement result in terms of the time required, the method considering all cases is not meaningful because it cannot be applied in the actual industry. Considering the method proposed in the present invention in terms of time required, it has an advantage over the existing method.

**Figure 8:**
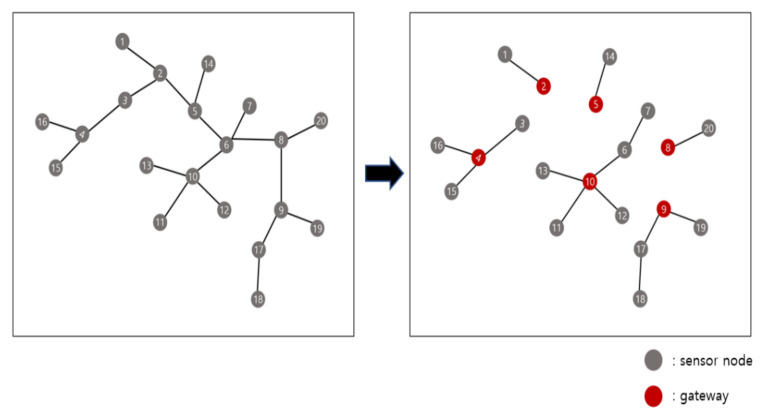
Example for 20 sensor nodes.

Figure 8 shows the results of applying the method proposed in this study to 20 sensor nodes. A total of six gateways are deployed, and it takes 21.19985 s to obtain the result for gateway deployment. The gateway deployment in this case is the same as that obtained by checking all cases.

**Figure 9:**
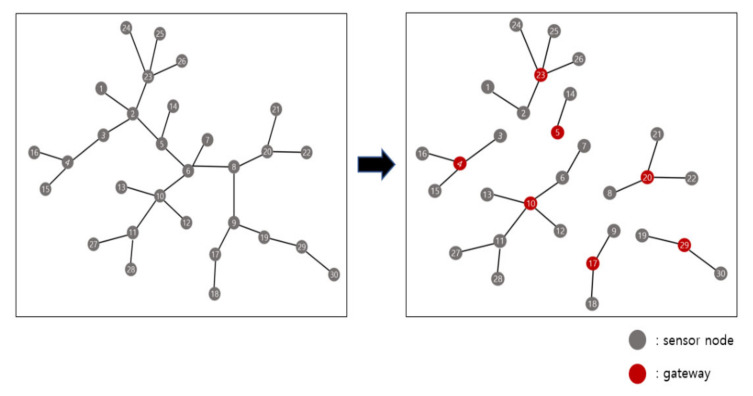
Example for 30 sensor nodes.

Figure 9 shows the results of applying the method proposed in this study to 30 sensor nodes. A total of six gateways are deployed, and it took 24.08383 s to obtain the result for gateway deployment. The gateway deployment in this case is the same as that obtained by checking all cases.

As a result, the proposed method spends time less compared to method that check all cases, but there is little difference in cost. The proposed method often produces the same results as method that checking all cases. Also, although not the most optimal result, the difference is very slight.

### 5.2. Comparison with the Genetic Algorithm

**Figure 10:**
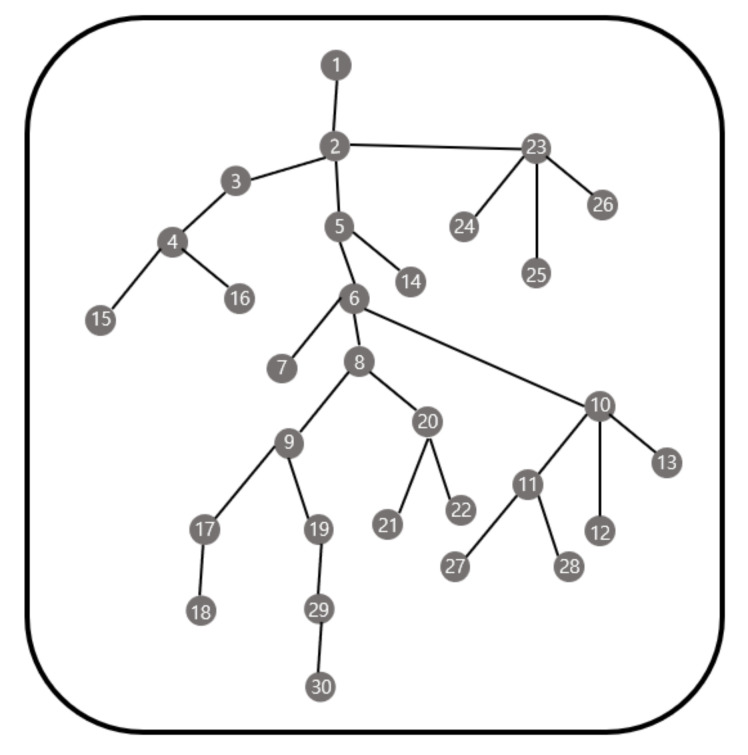
Topology of 30 sensor nodes.

**Figure 11:**
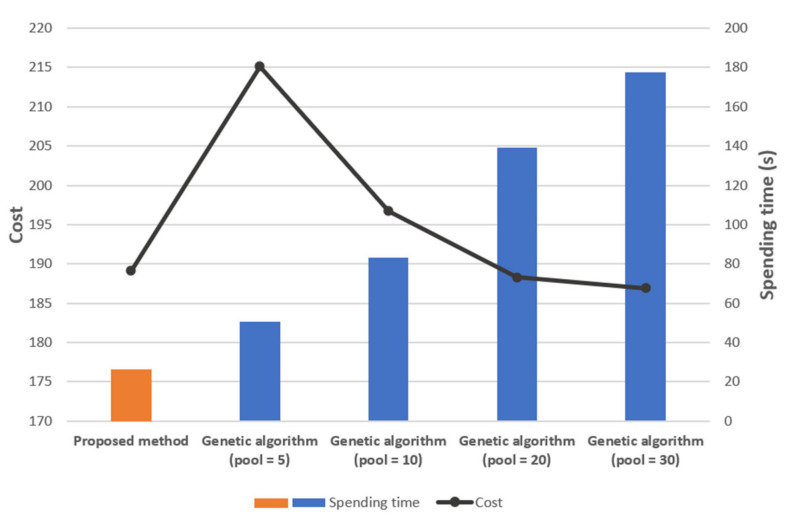
The comparison for the genetic algorithm (average for 5 times) and our methodology for 30 nodes topology in Figure 10**.**

We present a comparison with the genetic algorithm. Figure 10 is a topology of 30 sensor nodes. Figure 11 contains the results of numerical simulations using the genetic algorithm and our methodology for the topology in Figure 10. We use the parameters in (3) for the cost function. We also assume that if one gateway is responsible for more than half of the entire topology in the previous result, the gateway is additionally placed in the corresponding zone.

In the genetic algorithm, we set the crossover probability as 0.8 and the mutation probability as 0.006. The iteration number is 100. Since the genetic algorithm produces different results for the number of the initial pool, we experimented with increasing the number of pools such as 5, 10, 20, 30. The experiment was executed five times for each pool, and Figure 11 is based on the average value of the five simulations.

From these simulations, we observe that the cost of the deployment found by our methodology is lower than the cost of the deployment found by the genetic algorithm. Moreover, the computation time of our methodology is less than one of the genetic algorithms. We note that while our method always yields the same results, the genetic algorithm produces different results for each trial, since it depends on a random process and the given probabilities. Therefore, to obtain the optimal results for gateway deployment, it is necessary to iteratively experiment with adjusting the crossover and mutation probabilities. This process consumes a lot of time, but it does not guarantee that the deployment is optimal.

Moreover, in the genetic algorithm, it is difficult to consider the predetermined capacity of the gateway, which is the number of nodes that can be handled by each gateway. However, for each iteration in the genetic algorithm, the gateway is randomly chosen by the already determined parameters so that we cannot control the total number of sensor nodes that connect to one gateway. For example, we consider the topology in Figure 10 with the parameters in (3).

**Figure 12:**
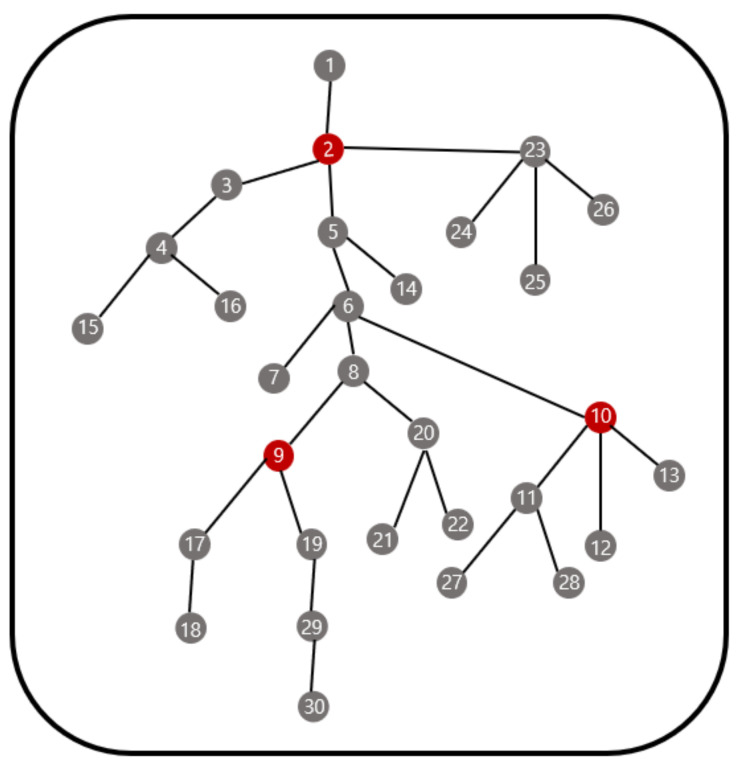
Gateway deployment by the genetic algorithm.

**Figure 13:**
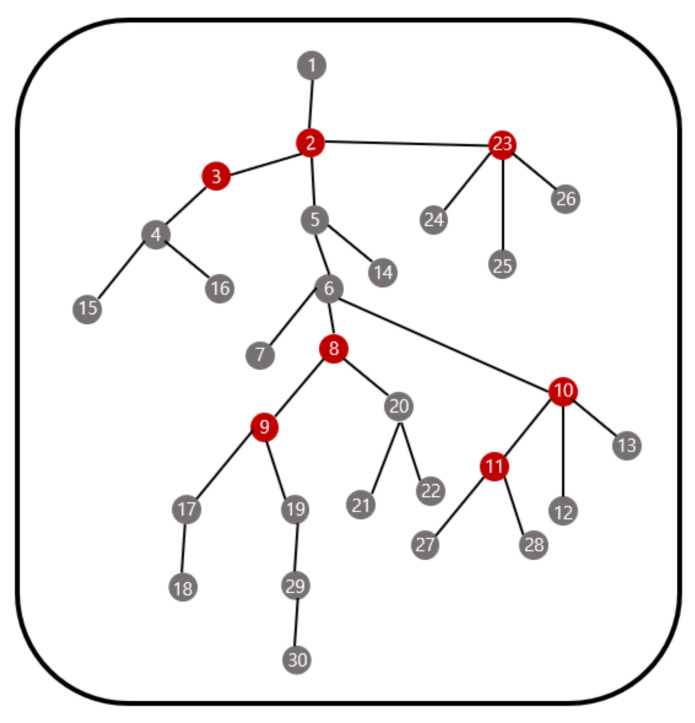
Gateway deployment by the proposed algorithm.

The genetic algorithm with the crossover probability = 0.8, the mutation probability = 0.006, the iteration number = 100, and the number of initial pools = 30 gives the gateway deployment in Figure 12. Since the capacity of the gateway in Figure 12 is 9 these three gateways cannot control all nodes of this network and this gateway deployment cannot be applied. On the other hand, our methodology produces the gateway deployment as shown in Figure 13 by applying the capacity which can be applied.

**Figure 14:**
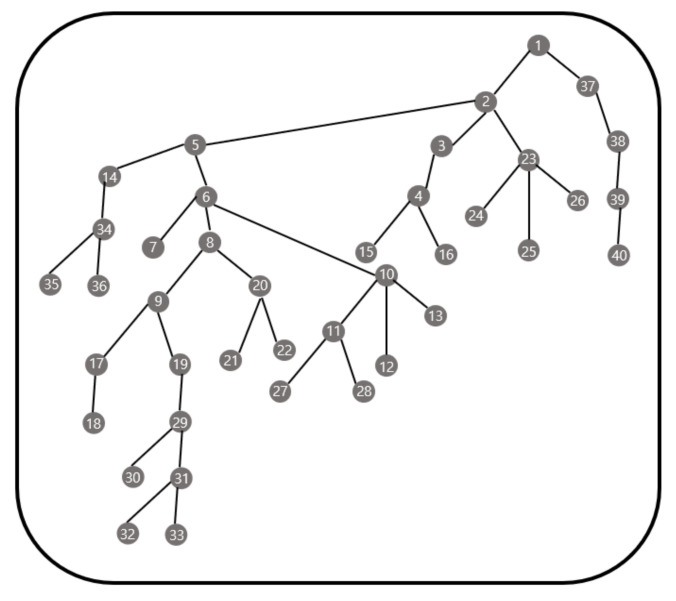
Topology of 40 sensor nodes.

**Figure 15:**
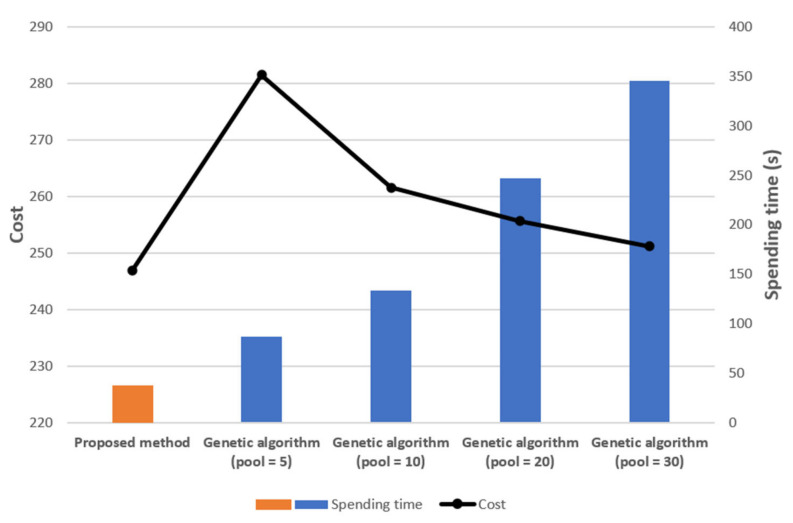
The comparison for the genetic algorithm and our methodology for 40 nodes topology in Figure 14**.**

We additionally provide a comparison of the genetic algorithm and our methodology for the 40 nodes topology in Figure 14. See Figure 15 for the comparison. We also set the crossover probability = 0.8, the mutation probability = 0.006, and the iteration number = 100 and we increase the number of pools as 5, 10, 20, 30 with the same cost function as in the previous case. The experiment was executed five times for each pool, and Figure 15 is based on the average value of five executions. For the genetic algorithm, when the number of the pools is small, the result may be limited because the result is produced by sampling from the pool. Therefore, we cannot obtain the lowest cost case. As the number of pools increases, the cost decreases, but the computation time increases. Because the combination of samples increases. On the contrary, the outcome of the proposed method is consistent.

## 6. Conclusions

In this paper, we propose a method of gateway deployment in WSNs. The proposed method is divided into four steps to arrange the gateway. With the divide and conquer method, the entire topology is divided based on the communication range, and the divided topology is divided once again based on the cost function. Then a gateway is added to areas where there are many connected sensor nodes. The method proposed in this paper is advantageous for large size networks due to the small amount of calculations needed. The main contribution of this study is to determine a gateway deployment that is close to the ideal gateway deployment in a short time. Additionally, it makes WSNs more cost-efficient. Moreover, the methodology in this paper would be combined with other stochastic algorithms described in Section 2 to obtain better results when considering the hybrid methodology. In addition, it is applicable to more general optimization problems on graphs of complex structures, such as two-tier problems. For example, there are hospitals with many wireless devices from patients and medical staff, as well as a power grid consisting of numerous sensors over a large area.

On the other hand, while our algorithm provides an efficient solution to the single-objective problem, its application to a multi-objective problem seems to require further research. We note that lifetime is regarded as an installation cost because sensors can be replaced in this paper. However, in a sensor-irreplaceable case, we should consider a multi-objective function that takes into account both lifetime and installation cost. Two-tier or measuring efficiency problem is also considered as a multi-objective problem. For these cases, more study is needed to apply a divide and conquer algorithm. Finally, in the case of the stochastic algorithm such as the genetic algorithm, there are degrees of freedom for generation and number of iterations, but our methodology lacks the degrees of freedom for problem solving. The proposed method is well established in most cases. However, we expect that the results could not sufficiently close to optimal if the network is extremely linear. These issues will be considered in our future research.

## Figures and Tables

**Table 1:** Average spending time to check all cases.

		The Number of Sensor Nodes							
		11	12	13	14	15	16	17	18
**Number of** **Simulation Runs**	**1**	45.33768	108.856	252.2552	588.867	1395.777	3346.086	7030.184	16088.71
	**2**	46.4142	108.5474	253.049	649.4481	1356.374	3065.544	6912.407	15885.37
	**3**	45.92477	106.6081	252.0893	630.1877	1403.543	3412.523	7123.94	15923.11
	**4**	45.42486	107.6882	256.8998	639.413	1389.235	3387.412	7111.85	15488.22
	**5**	45.09183	108.1149	258.3735	640.1325	1394.432	3314.324	6899.523	16183.2
**Average(s)**		45.63867	107.9629	254.5334	629.6096	1387.872	3305.178	7015.581	15913.72

**Table 2:** Average spending time for the proposed method.

		The Number of Sensor Nodes							
		11	12	13	14	15	16	17	18
**Number of** **Simulation Runs**	**1**	0.695669	1.851832	4.0123	5.398969	5.567255	7.838274	11.45018	14.1234
	**2**	0.489427	1.917904	3.221347	5.350707	6.392298	8.11234	12.4214	14.1256
	**3**	0.554424	2.062249	3.7213	6.063138	6.109328	7.9934	12.15124	15.92189
	**4**	0.431641	1.973217	4.248372	5.23412	7.1143	8.0124	11.94673	15.1235
	**5**	0.585471	1.734261	3.612864	5.28442	6.9932	8.412354	10.93876	15.7315
**Average(s)**		0.551326	0.551326	1.907893	3.763237	5.466271	6.435276	8.073754	11.78166

## Data Availability

Not applicable.
